# Post-Recession Housing Insecurity and Physical and Mental Health of Midlife and Aging Adults

**DOI:** 10.1093/geroni/igab046.2873

**Published:** 2021-12-17

**Authors:** Aarti Bhat, David Almeida, Alexis Santos

**Affiliations:** 1 Pennsylvania State University, State College, Pennsylvania, United States; 2 Pennsylvania State University, University Park, Pennsylvania, United States

## Abstract

Housing insecurity, or limited/unreliable access to quality housing, is a powerful chronic stressor that can negatively affect individual health and well-being. This study extends prior research by examining the effect of multiple forms of housing insecurity on both the mental and physical health of aging adults using the Midlife in the United States study (MIDUS; N = 2532; M age = 63.42; 57% women; 16% black). Participants reported on experiences of anxiety/depression in the past year, number of chronic health conditions experienced in the last year, and experiences of housing insecurity since the 2008 recession (e.g., homelessness, threatened with foreclosure or eviction, missed rent or mortgage payment). 14% of participants reported experiencing one or more housing insecurity events in the aftermath of the recession. Higher levels of housing insecurity were experienced by midlife participants (ages 46-65) and black participants. Regression results showed that, even when controlling for prior health, housing insecurity was significantly associated with higher odds of experiencing anxiety/depression and additional chronic health conditions. These results suggest that housing insecurity experiences are fairly prevalent among midlife and aging adults, and that housing insecurity experiences leave these adults susceptible to compromised mental and physical health. This work has various implications for policy around addressing housing access and affordability issues for aging adults as a public health concern. Subsequent analyses will examine age, gender, and race/ethnic differences in these associations between housing insecurity and health outcomes.

